# Extraction and Detection of Structurally Diverse Siderophores in Soil

**DOI:** 10.3389/fmicb.2020.581508

**Published:** 2020-09-17

**Authors:** Vineeta Rai, Nathaniel Fisher, Owen W. Duckworth, Oliver Baars

**Affiliations:** ^1^Department of Entomology and Plant Pathology, North Carolina State University, Raleigh, NC, United States; ^2^Department of Ecology and Evolutionary Biology, Cornell University, Ithaca, NY, United States; ^3^Department of Crop and Soil Sciences, North Carolina State University, Raleigh, NC, United States

**Keywords:** siderophores, extraction, recovery, iron, soil, adsorption, α-hydroxycarboxylate, catecholate

## Abstract

Although the biochemistry of bacterial and fungal siderophores has been intensively studied in laboratory cultures, their distribution and impacts on nutrient cycling and microbial communities in soils remain poorly understood. The detection of siderophores in soil is an analytical challenge because of the complexity of the soil matrix and their structural diversity. Liquid chromatography-mass spectrometry (LC-MS) is a suitable method for the sensitive analysis of siderophores in complex samples; however, siderophore extraction into liquid phases for analysis by LC-MS is problematic because of their adsorption to soil particles and organic matter. To determine extraction efficiencies of structurally diverse siderophores, spike-recovery experiments were set up with standards representing the three main siderophore classes: the hydroxamate desferrioxamine B (DFOB), the α-hydroxycarboxylate rhizoferrin, and the catecholate protochelin. Previously used solvent extractions with water or methanol recovered only a small fraction (< 35%) of siderophores, including < 5% for rhizoferrin and protochelin. We designed combinatorial chemical extractions (22 total solutions) to target siderophores associated with different soil components. A combination of calcium chloride and ascorbate achieved high and, for some soils, quantitative extraction of DFOB and rhizoferrin. Protochelin analysis was complicated by potential fast oxidation and interactions with colloidal soil components. Using the optimized extraction method, we detected α-hydroxycarboxylate type siderophores (viz. rhizoferrin, vibrioferrin, and aerobactin) in soil for the first time. Concentrations reached 461 pmol g^–1^, exceeding previously reported concentrations of siderophores in soil and suggesting a yet unrecognized importance of α-hydroxycarboxylate siderophores for biological interactions and biogeochemical processes in soil.

## Introduction

Siderophores are produced by bacteria, fungi, and graminaceous plants to promote the chelation and uptake of the trace-nutrient iron (Fe). Siderophores are secondary metabolites (molecular weight < 1500 Da) that are secreted into the extracellular environment where they chelate ferric iron (Fe^III^) with high affinity. The Fe(III)-siderophore complexes are available to the cell via uptake by specific receptors, or by cell-surface reduction and uptake of Fe(II). Because they affect the availability of Fe, which can potentially limit microbial growth, siderophores play critical roles in host-microbe and microbiome interactions, including ‘tug-of-wars for iron’, ‘siderophore cheating’, and plant-growth promoting mechanisms (Haas et al., 2008; Swinburne, 2012; Butaitė et al., 2017).

Siderophores have been intensively studied and reviewed from the perspective of their chemistry and biochemistry (Hider and Kong, 2010), soil chemistry (Crowley and Kraemer, 2008), plant pathology (Swinburne, 2012), host-pathogen interactions, and microbiome community interactions (Kramer et al., 2019). Siderophore production is induced by low intracellular iron concentrations, which are linked to iron bioavailability in the medium (Colombo et al., 2014). They provide a competitive growth advantage under conditions when the total iron concentration in the medium is replete, but the fraction of bio-available iron is low. Such conditions may be found in a biological host (Kramer et al., 2019) and in aerobic soil (Colombo et al., 2014).

Most of the insight on the biological importance of siderophores has been gained using laboratory incubations with media that allow for control of iron availability and facilitate analysis of siderophores. Siderophore concentrations secreted into the surrounding medium are typically in the μM range but can even reach mM concentrations in highly active liquid laboratory cultures. A large fraction of soil-dwelling bacteria and fungi and graminaceous plants have been shown to produce siderophores, based on analyses of secreted metabolites in culture or the presence of siderophore biosynthesis genes (Hider and Kong, 2010). Based on this evidence, it is generally accepted that siderophores play a critical role in the acquisition of iron and other micronutrients by plant and soil microbes. Yet, because of analytical difficulties, there is limited direct evidence of siderophores in soils, and there are significant questions regarding where and when siderophore production becomes critical to organism nutrition, microbial competition, or soil biogeochemical processes. A better understanding of siderophore production and distribution in soil will help to elucidate the role of iron as a bottom-up control on microbial activity, microbiome interactions, and plant growth.

Methods using liquid chromatography-mass spectrometry (LC-MS) have enabled the profiling of siderophores in complex matrixes (Boiteau et al., 2013; Baars et al., 2014; Deicke et al., 2014). However, a major difficulty is that siderophores (and other metabolites) can strongly adsorb to soil particles. Such adsorption depends on the structure of the siderophore and may prevent the extraction of some siderophores into liquid phases for LC-MS analysis. Only a handful of studies have attempted direct analyses of siderophores in soil (Essen et al., 2006; Ali et al., 2011; Ahmed and Holmstrom, 2014; Schenkeveld et al., 2014a; Boiteau et al., 2018). Previous methods used water and methanol for extraction and detection by LC-MS. Ali et al. (2011) also used K_2_HPO_4_ (1 mM, pH = 7.5) as a buffer. These studies demonstrate the widespread presence of siderophores in soil. Interestingly, all of the reported studies detected hydroxamates, but the two other major classes of siderophores, α-hydroxycarboxylates and catecholates, have not been targeted or detected.

The goal of this study was to develop extraction strategies for siderophores, determine the extraction efficiencies for structurally diverse siderophores in soils, and survey for bacterial and fungal siderophores in soils with varying edaphic properties. Spike recovery experiments were performed with three standards representing the major siderophore types and chemistries: the positively charged tris-hydroxamate desferrioxamine B (DFOB) (Dhungana et al., 2001), the hydrophilic negatively charged α-hydroxycarboxylate rhizoferrin (Carrano et al., 1996) and the neutral or negatively charged hydrophobic tris-catecholate protochelin (Harrington et al., 2012b). The results showed that frequently used water and methanol extractions recover only minor fractions of the three siderophores, particularly in the case of rhizoferrin and protochelin (< 5%). Chemical extraction methods (22 different solutions) were systematically designed to minimize specific adsorption, electrostatic interactions, and hydrophobic interactions. High recoveries for the hydroxamate DFOB and the α-hydroxycarboxylate rhizoferrin were achieved by aqueous extraction with ascorbic acid and Ca^2+^ followed by re-extraction with methanol. Analyses of the catecholate siderophore protochelin were complicated in all conditions due to interactions with co-extracted colloidal soil components, which reduced chromatographic peaks, and potential fast oxidation reactions of the catecholate in soil. Application of the extraction method suggested that α-hydroxycarboxylate siderophores, observed here for the first time in soil samples, may be common in the upper soil layer.

## Materials and Methods

### Soil Sample Collection

Soil samples were collected at nine locations across North Carolina in the summer and fall of 2018 (see Supplementary Table S1 for location, pH, and other soil properties). Sampling sites were selected for their contrasting soil edaphic properties (e.g., high-organics, loamy, sandy). The O- or A- horizon of these samples was sieved (2 mm mesh) and stored at 4°C. One soil sample (S2; O-horizon) was selected to determine and optimize the recoveries with 22 tested chemical extraction solutions (see section “Chemical Extractions”). This sample was collected near the laboratory (Raleigh, North Carolina). To determine the effect of soil edaphic properties on recoveries, the optimized extraction method was applied to six additional samples (S11, S21, S51, S71, S81, S91; A-horizon). Finally, the optimized extraction method was used to survey for the presence of siderophores in each of these samples (S1, S11, S21, S51, S71, S81, S91) and in two additional samples collected in Raleigh, North Carolina (S1, S3; O-horizon).

### Siderophore Spiking

**FIGURE 1:**
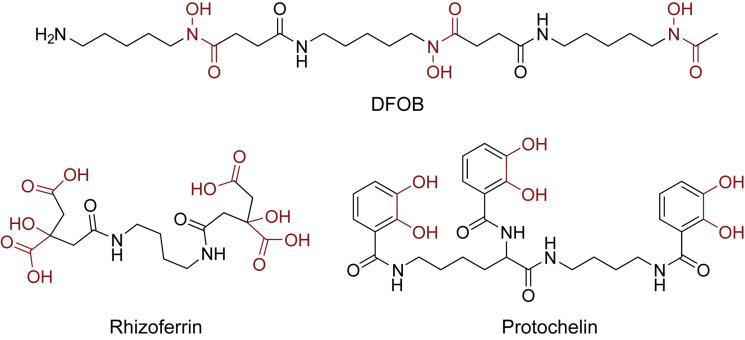
Structures of the three siderophore standards used in this study: the hydroxamate siderophore desferrioxamine B (DFOB), the α-hydroxycarboxylate siderophore rhizoferrin, and the catecholate siderophore protochelin. The iron chelating moieties are indicated in red.

Soil samples (0.5 g) were spiked with an aqueous siderophore standard mixture (0.5 mL) in conical centrifuge tubes (Falcon, 15 mL). The siderophore standard mixture contained 6.25 μM of each of the three standards desferrioxamine B (DFOB, Sigma Aldrich), rhizoferrin, and protochelin ([DFOB] = 6.25 μM; [rhizoferrin] = 6.25 μM; [protochelin] = 6.25 μM) (Figure 1). Rhizoferrin and protochelin were synthetically prepared in sufficient amounts by the Small Molecule Synthesis Facility (SMSF) at Duke University (Harrington et al., 2012a). The soil sample with the siderophore mixture was equilibrated overnight at room temperature in the dark and lyophilized to obtain spiked soil samples containing a concentration of 6.25 ng g^–1^ of each siderophore.

### Chemical Extractions

Siderophore recoveries were determined with 22 chemical extraction solutions (Table 1) using the same soil sample (S2) that was spiked prior to each extraction. Duplicate extractions were set up for each of the tested solutions. The extractions proceeded by addition of a volume of 3 mL of the selected extraction solution to the lyophilized spiked sample. The sample was then adjusted to pH = 7 unless otherwise noted (Table 1). Extractions were assisted by overnight agitation on a 3-dimensional orbital shaker, followed by ultrasonication (Fisherbrand CPXH) for 15 min. Soil pellet and supernatant were separated by centrifugation, and the pellet was sequentially re-extracted with 5%, 25%, and 80% methanol (3 mL of each) by ultra-sonication for 15 minutes (except in those experiments where a single solvent was tested, Table 1). The 5%, 25%, and 80% methanol solutions did not include any additional extraction reagents. The supernatants from the extractions were pooled, evaporated to dryness (Thermo SpeedVac), reconstituted in 0.5 mL of 50% methanol, and filtered (Millex GP, 0.2 μm). Extracted samples were stored at 4°C until analysis by LC-MS within one week.

**TABLE 1:** Extraction solutions

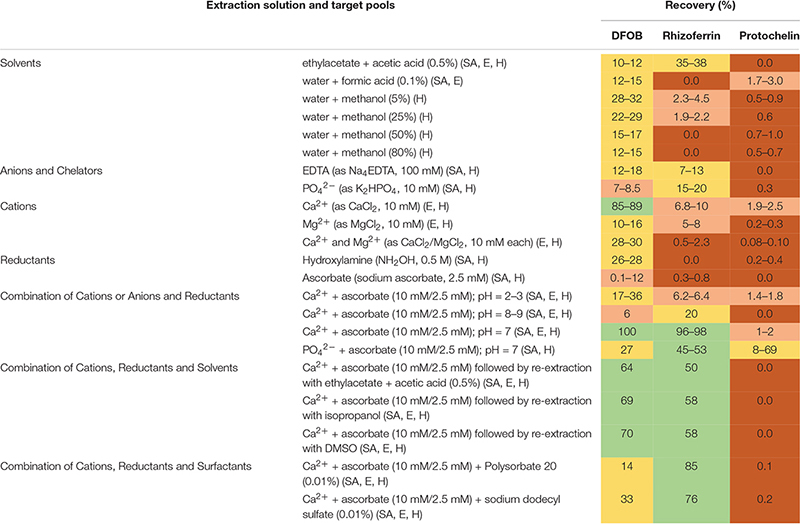

* all extractions were done in duplicates except where no range is given. Unless specifically stated, extraction agents were dissolved in water and adjusted to pH = 7. After extraction with a given extraction solution, samples were sequentially re-extracted with 5, 25, and 80% methanol (except for those conditions where a specific solvent was tested – ‘Solvents’ conditions). The four extracts were pooled for analysis of recoveries. SA, Specifically Adsorbed; E, Electrostatic Interactions; H, Hydrophobic Interactions. Colors indicate high (> 50%, green), intermediate (10–50%, yellow), low (1–10%, rose), and negligible (<1%, brown) recoveries.

The optimized extraction procedure consisted of extraction with an aqueous solution of 10 mM CaCl_2_ and 2.5 mM sodium ascorbate at pH = 7 followed by sequential re-extraction with 5, 25, and 80% methanol solutions. Rhizoferrin and DFOB were detected predominantly in the aqueous and 5% methanol fractions (∼90% of the combined signal). Protochelin was distributed between the aqueous/5% methanol fractions and the 25/80% methanol fractions.

### Siderophore Quantification by Liquid Chromatography – Mass Spectrometry

The soil extracts (in 50% methanol as described above) were acidified with formic acid for analysis by LC-MS (Ultimate 3000 UPLC/ISQ EC, Thermo Fisher Scientific). The instrument was also equipped with a UV/vis Diode Array Detector (DAD) and a Charged Aerosol Detector (CAD). A volume of 100 μL was injected and separated on a C18 column (Agilent Poroshell EC-C18, 2.7 μm, 4.6 × 100 mm) with matching guard column using a gradient of solutions A and B (0% B for 1.5 min, then 0–100% over 7.5 min, constant at 100% until 10 min; A: water + 1% acetonitrile + 0.1% formic acid, B: acetonitrile + 2% water + 0.1% formic acid; flow-rate 1.2 ml/min; temperature 30°C). The method included an on-line desalting step by diverting the flow to waste for the first 3 min during which added salts or hydrophilic chemical extraction agents eluted. The on-line desalting step eliminated potential analyte loss during sample preparation steps (e.g., during solid-phase extraction). The mass spectrometer was set to cycle through target *m/z* values in Single Ion Monitoring mode (SIM). The cycle also included full-scans (m/z = 149–1250) in positive and negative modes. The SIM ion list included: apo-DFOB (positive mode, *m/z* = 560.4), DFOB-Fe (positive mode, *m/z* = 614.3), apo-rhizoferrin (negative mode, *m/z* = 435.1), and apo-protochelin (negative mode, *m/z* = 623.2). To simplify the annotation, in the following, we discuss the recovery of DFOB as the summed recoveries of apo-DFOB and DFOB-Fe. The Fe complexes of rhizoferrin and protochelin were negligible at the mobile phase pH (pH < 3; 0.1% formic acid), even in an excess of iron. This was in agreement with the expected chemistry and previous observations that α-hydroxycarboxylate and catecholate siderophores are unstable and dissociate quickly upon acidification (Hider and Kong, 2010; Baars et al., 2014). Siderophores were identified by a match of retention time and *m/z*. Quantification for the evaluation of the 22 different extraction solutions was achieved by external standard calibration using standards added to a blank soil extract matrix (using sample S2). For evaluation of recoveries with different soil types, quantification involved internal standard addition calibration to account for different soil extract matrix effects with different soil types (samples S11, S21, S51, S71, S81, S91). After measurement of the extracted soil sample, known concentrations of the DFOB/rhizoferrin/protochelin standard mixture were added to the extracted sample. Two internal standard additions were done in this way to generate an internal standard calibration curve. To reduce metal contamination of the column, a pH = 6 solution of 0.1 mM ethylenediaminetetraacetate (EDTA) was passed over the HPLC column at the end of the day and run overnight at a flow rate of 0.4 mL min^–1^.

### Analysis of Unspiked Soil Samples

All nine soil samples were surveyed for the presence of siderophores using the optimized method for extraction but increasing the soil sample amount from 0.5 g to 3 g to enhance LC-MS signals of siderophores present at lower concentrations. At the same time, extraction solution volumes were increased from 3 mL to 5 mL: An aqueous solution containing 10 mM CaCl_2_ and 2.5 mM sodium ascorbate (5 mL) was added to soil (3 g) at pH = 7. The sample was extracted overnight at room temperature in the dark on a 3-dimensional orbital shaker followed by ultra-sonication for 15 min (Fisherbrand CPXH) and centrifugation. The soil pellet was sequentially re-extracted with 5, 25, and 80% methanol (5 mL of each without additional reagents) by ultrasonication for 15 min. The supernatants from these four extractions were pooled, evaporated to dryness (Thermo SpeedVac), reconstituted in 0.5 mL 50% methanol, and filtered (Millex GP, 0.2 μm). Nine siderophore standards (DFOB, rhizoferrin, protochelin, aerobactin, vibrioferrin, amphibactin ACA, azotochelin, desferrioxamine E, and pyoverdine 7.1) were used for targeted analysis of siderophores in the extracts by a match of retention time and *m/z*. The ionization modes and *m/z* values for each standard are given in Supplementary Table S2. Rhizoferrin was the most concentrated siderophore in our survey of soil samples. Internal standard calibrations were set up in the case of rhizoferrin to achieve accurate quantification: after measurement of the extracted unspiked soil sample, known concentrations of a rhizoferrin standard were added to the extracted sample. Four internal standard additions were done in this way to generate an internal standard calibration curve.

### Effect of Co-extracted Colloidal Components on LC-MS Peak Areas

The effect of co-extracted colloidal (< 0.2 μm) soil components on LC-MS analysis results was determined to elucidate the cause of low protochelin peaks in LC-MS analyses. Unspiked soil (sample S2) was extracted with the optimized extraction method (see above) and filtered (0.2 μm Millex GP). The extracts were then ultrafiltered through 3-kDa cartridges (Amicon Ultra, Millipore) and compared to controls that were not ultrafiltered. Siderophore standards were added before or after ultrafiltration. Tests were done to elucidate if solid-phase-extraction (SPE) clean-up methods could reduce the effect of co-extracted colloidal components on protochelin peaks. Unspiked soil samples were extracted with the optimized extraction method and siderophore standards were added. These samples were purified by SPE (Oasis HLB columns, activated with methanol; conditioning with 0.1% formic acid; loading of sample acidified with 0.1% formic acid; cleaning with 0.1% formic acid; elution with methanol) before LC-MS analysis.

### Procedure for Extraction Under Low Oxygen Conditions

Catechol siderophores, such as protochelin, are prone to oxidation (Devlin and Harris, 1984; Harrington et al., 2012a). To determine the potential oxidation of siderophores during the spiking or extraction procedure, several spike-recovery experiments were set up under low oxygen conditions. Soil samples (sample S2) were spiked under exclusion of oxygen by purging the soil-spike solution (0.5 g soil, 0.5 mL spike solution) with N_2_ (15 min) in Balch tubes. Tubes were then closed with rubber stoppers and crimped. Spiked soil samples were vortexed for 15 min, frozen with liquid N_2_ and lyophilized. Subsequent extractions proceeded by using N_2_-purged solutions in Balch tubes. For LC-MS analysis, samples were transferred under a N_2_-stream to autosampler vials and analyzed immediately. To evaluate the effect of redox-active metals on analysis results N_2_-purged FeCl_3_ or MnCl_2_ standards were prepared and added to the soil extracts before LC-MS analysis.

### Soil Chemical and Physical Properties

Samples for the analysis of water extractable metal concentrations were prepared by agitating soil samples (1 g) with 10 ml of 18.2 MΩ cm water (MQ water) with a 3-dimensional rotary shaker for an hour at room temperature. Samples were filtered with syringe filters (Millipore Millex GP 0.22 μm) before acidification (2% HNO_3_) and addition of internal standards (Sc, 5 ppm; In, 10 ppm). Samples were diluted (1:4) with 2% HNO_3_ for analysis by inductively coupled plasma−mass spectrometry (Thermo iCAP−RQ in KED mode). Metal concentrations in the soil water extracts (including ^55^Mn, ^56^Fe, ^27^Al) were quantified by external standard calibrations run at the beginning and end of the sample queue.

Total iron content of soil, % organic matter, cation exchange capacity (CEC), and soil particle sizes were analyzed by the Environmental and Agricultural Testing Service Laboratory (EATS) at North Carolina State University. Iron content was analyzed after acid digestion according to EPA Method 3050b by ICP-OES (Perkin Elmer 8000). Organic matter was determined as a percent fraction of the soil by subtracting the dry soil weight from the ‘ash weight’ and dividing by the dry weight. The dry weight was determined by drying in a 105°C oven for 24 h. ‘Ash’ weight was determined after placing the dried soil in a muffle furnace at 400°C for 16 h. CEC was determined by displacement of cations with a 1 M ammonium acetate solution (pH = 7) using 95% ethanol as rinsing solution, and replacement of adsorbed ammonium with 1 M potassium chloride. Exchangeable ammonium in the KCl solution was determined by the Lachat colorimetric analysis. Soil particle sizes were analyzed with a hydrometer (Gee and Or, 2002).

## Results and Discussion

### Formulation of Chemical Extraction Solutions

Siderophores can strongly adsorb to soil particles and colloids (Romheld, 1991; Ahmed and Holmstrom, 2014; Harrington et al., 2015), and a significant number of studies have sought to better understand what specific components of soils interact with siderophores and Fe-siderophore complexes. It is well established that siderophores specifically adsorb to metal oxide and hydroxide surfaces (Holmen and Casey, 1996; Holmen et al., 1997; Cocozza et al., 2002; Cheah et al., 2003; Duckworth and Sposito, 2005; Upritchard et al., 2007; Duckworth et al., 2008, 2014). Additionally, previous studies have shown that the positively charged siderophore DFOB and DFOB-metal complexes may adsorb to negatively charged surfaces or intercalate into clay minerals, indicative of electrostatic interactions (Neubauer et al., 2000; Siebner-Freibach et al., 2004, 2006; Maurice et al., 2009; Mishra et al., 2010). Catechol siderophores have been found to strongly adhere to mineral surfaces by hydrogen and coulomb bonding (Maier et al., 2015) or coordination on metal oxides (Saiz-Poseu et al., 2019). Apart from specific absorption or electrostatic interactions, soil may sequester siderophores and their Fe^3+^ complexes by hydrophobic interactions. For example, DFOB and DFOB-metal complexes can be associated with organic matter via hydrophobic partitioning (Solinas, 1994; Higashi et al., 1998). Catechol moieties are also well known for their hydrophobic character (Saiz-Poseu et al., 2019). These adsorption mechanisms (i.e., specific adsorption, electrostatic interactions, hydrophobic partitioning) may combine to strongly retain siderophores. For example, all three interactions can contribute during cation bridging of DFOB and DFOB-metal complexes to organic matter (Solinas, 1994; Higashi et al., 1998).

To profile and quantify siderophores in soil, extraction solutions must be able to release sequestered siderophores adsorbed to soil particles by any of these different mechanisms. We thus designed chemical extractions to:

(1) solubilize specifically adsorbed (**SA**) siderophores by competition for adsorption sites, or by concomitant release associated with the dissolution of minerals (e.g., by reduction of Fe^3+^ to Fe^2+^). Chelating agents and reductants may target the SA pool. Acidification may dissociate specifically adsorbed siderophore metal complexes that are pH labile.
(2) release siderophores or siderophore complexes held by electrostatic absorption (**E**). Divalent cations may exchange siderophores held by electrostatic interactions with cation-exchange sites. Acidification can protonate cation exchange sites or negatively charged siderophores. Basic extraction solutions may neutralize anion exchange sites or positively charged siderophores.
(3) desorb siderophores or siderophore complexes retained by hydrophobic interactions (**H**). Low-polarity solvents or surfactants can solubilize compounds retained by hydrophobic interactions.

In total, we evaluated 22 extraction solutions (Table 1). It is worth noting that extractants often target more than one pool.

### Synergistic Extraction Efficiency With Ca^2+^ and Ascorbate

**FIGURE 2:**
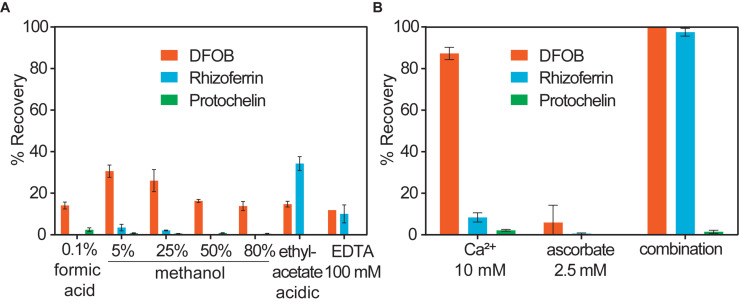
Soil siderophore spike-recovery for DFOB, rhizoferrin, and protochelin (each spiked at 6.25 nmol g^–1^). **(A)** Solvent extractions show low recoveries, particularly for rhizoferrin and protochelin. **(B)** A strong synergistic effect was observed for DFOB and rhizoferrin recovery when Ca^2+^ and ascorbate were used in combination as chemical extraction agents.

Water or methanol were previously used for extraction of siderophores from soil (Essen et al., 2006; Ahmed and Holmstrom, 2014; Boiteau et al., 2018). In our extractions of siderophore spiked soil, water or methanol extractions recovered only a small fraction of siderophores (< 35%), particularly in the case of rhizoferrin and protochelin (< 5%) (Table 1 and Figure 2A). Acidified ethyl acetate (0.5% acetic acid) enhanced the recovery of rhizoferrin (∼35%), but reduced DFOB recovery (∼11%), and protochelin was not extracted. These observations suggested that hydrophobic interactions (H) were not the principal reason for low recoveries.

Electrostatic interactions (E) may be reduced by the addition of divalent cations (Ca^2+^ or Mg^2+^), which saturate particulate cation exchange sites to which siderophores can bind (Maurice et al., 2009). Indeed, addition of Ca^2+^ (as CaCl_2_, 10 mM) to extractants increased the recovery of the cationic DFOB to more than 80% (Table 1 and Figure 2B). We also evaluated Mg^2+^, but it recovered only a low amount of DFOB. An explanation for this differential extraction efficiency may be that Ca^2+^ binds significantly stronger to clay and humic matter than Mg^2+^ (Rytwo et al., 1996). No effect of Ca^2+^ or Mg^2+^ was observed for the extraction of the negatively charged rhizoferrin or protochelin (Table 1 and Figure 2B).

The use of EDTA or phosphate in extraction solutions may chelate Fe^3+^ and other metals, dissociate clay micelles, and thus dissolve specifically adsorbed siderophores (SA). However, these extractions did not show high recoveries of DFOB or rhizoferrin (< 20% recoveries) and very low recovery of protochelin. We then evaluated reductants (hydroxylamine, ascorbate) to reduce minerals to which siderophores may specifically adsorb. Reductants may also stabilize siderophores that are susceptible to oxidation. Ascorbate has an additional function as a chelating agent for Fe^2+^ and Fe^3+^ (Conrad and Schade, 1968). Using hydroxylamine (NH_2_OH) or sodium ascorbate, we found only low recoveries for DFOB, and no significant recovery for rhizoferrin and protochelin (Table 1 and Figure 2B).

In further attempts to increase recoveries, we evaluated combinations of the above reagents. A strong synergistic effect was observed for Ca^2+^ in conjunction with ascorbate. A combination of the two reagents fully recovered both, DFOB and rhizoferrin, at neutral pH (Table 1 and Figure 2B). Based on the cumulative results of the extractions, SA and E mechanisms were thus considered likely critical for increasing siderophore recoveries. However, the measured protochelin recovery remained low in all cases. Changing the pH of the extraction solutions to acidic (pH = 2–3) or mildly basic (pH = 8–9) significantly reduced the recoveries of all siderophores. The concentrations of Ca^2+^ (10 mM) and ascorbate (2.5 mM) were selected to achieve near quantitative recovery, and higher concentrations did not have a further beneficial effect in our experiments. The concentration of Ca^2+^ (equivalent to 12 cmol_c_ kg^–1^ soil) exceeded the CEC of the soils used in this study (Supplementary Table S1). Addition of surfactants or solvents to the extraction mixtures to counter hydrophobic interactions, which may increase recovery of the hydrophobic protochelin, did not show a significant positive effect. Reasons for the low protochelin signals are evaluated and discussed below.

### Causes of Low Recoveries of the Catechol Siderophore Protochelin

**FIGURE 3:**
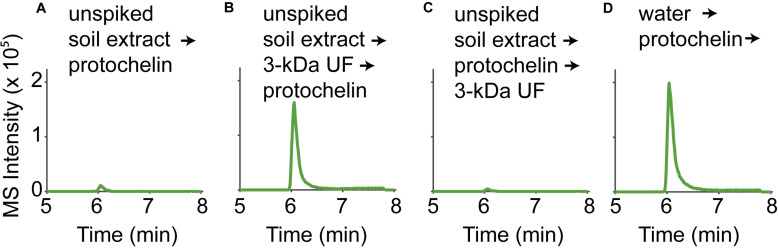
LC-MS signals of a protochelin standard (3.1 μM) in **(A)** an unspiked soil extract; **(B)** an unspiked soil extract that was 3-kDa ultrafiltered before protochelin addition; **(C)** an unspiked soil extract that was 3-kDa ultrafiltered after protochelin addition; **(D)** water.

All spiked soil extracts showed low protochelin recoveries. In the following, we examine potential interactions of protochelin with soil components that prevented its recovery or detection. In addition, these studies provided insight into interactions of DFOB and rhizoferrin with soil. Quantification of the siderophores was based on LC-MS analysis using an external standard calibration with standards dissolved in an extract of unspiked soil. The calibration of standards dissolved in an unspiked soil extract (instead of standards dissolved in water) was important to correct for matrix effects caused by co-extracted soil components, such as co-extracted organic matter. Matrix effects were pronounced for protochelin: protochelin standards added to unspiked soil extracts had much lower LC-MS peak areas than the same standards dissolved in water (∼10x reduced peak area, Figure 3, panels A and D). In contrast to protochelin, the analytical signals for DFOB and rhizoferrin were not reduced in the soil extract matrix. Because unspiked soil extract was used for the calibration, matrix effects were accounted for during calculation of extraction recoveries. However, insight into which co-extracted soil components were responsible for the matrix effect may provide insight into protochelin interactions with soil and to better understand how quantitative extraction and sensitive detection may be achieved. It is noteworthy that protochelin and other catechol siderophores have distinct chemical properties (e.g., redox reactivity, hydrophobicity, complexation, and adsorption affinities) and that, to our knowledge, these siderophores have not previously been detected in any environmental samples.

An immediate consideration was that ion suppression effects in electrospray ionization with LC-MS could have caused lower protochelin signals. One way to detect if ion suppression was significant was to compare MS peak areas to those obtained by other HPLC detectors. A Charged Aerosol Detector (CAD) and UV detector (protochelin catechol absorption at 315 nm) were connected to the same HPLC system. All three detectors showed good correlation (low protochelin signals) when the standard was dissolved in unspiked soil matrix extracts indicating that ion suppression effects were not the cause of the low protochelin peaks. Next, we considered matrix effects related to co-extracted soil components as the cause of low protochelin signals: (1) dissolved colloidal components in the soil extract, such as humic matter or clay interacted with protochelin leading to reduced LC-MS signals or (2) dissolved metals affected siderophore speciation. Finally, it was tested, if (3) the presence of oxygen caused quick oxidation of protochelin after it was spiked into a soil sample.

#### Protochelin Interactions With Colloidal Soil Components

The influence of colloids in soil extracts was evaluated using 3-kDa ultrafiltration cartridges to remove colloids from filtered (< 0.2 μm) unspiked soil extract solutions (Figure 3). Signals for protochelin were significantly higher in 3-kDa ultrafiltered unspiked soil extracts and comparable to the signal of a protochelin standard dissolved in water (Figures 3A,B,D). These results indicated that co-extracted colloids were primarily responsible for the lower protochelin signals. The next question was if ultrafiltration of a protochelin standard dissolved in a filtered (< 0.2 μm), unspiked soil extract would selectively remove colloidal components and increase protochelin signals or if protochelin would be removed from the solution along with colloids. We observed that only 2% of protochelin remained in solution after ultrafiltration (Figure 3C), so that this approach was not suited to improve quantification. We attempted purification of soil extracts by solid-phase-extraction (SPE) to selectively remove colloids. Using reversed phase SPE (Oasis HLB), protochelin was not detectable in the flow-through or any eluate fractions. Together, these results indicate strong interactions of protochelin with colloids, which were the main explanation for matrix effects during LC-MS detection. We suspect that similar interactions of protochelin with colloidal soil particles are also a critical factor for the low extraction efficiencies.

#### Interaction With Dissolved Metals

**FIGURE 4:**
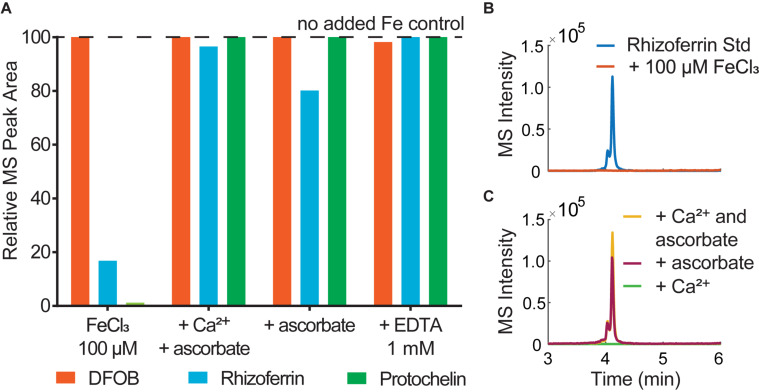
**(A)** Analysis of siderophore standards (6.25 μM) in water immediately after addition of (1) 100 μM FeCl_3_; (2) 100 μM FeCl_3_, 10 mM CaCl_2_, 2.5 mM ascorbate; (3) 100 μM FeCl_3_, 2.5 mM ascorbate, (4) 100 μM FeCl_3_, 1mM EDTA. **(B)** LC-MS peak of a rhizoferrin standard (3.1 μM) in water and comparison to the same standard after addition of 100 μM FeCl_3_. **(C)** LC-MS signals of 3.1 μM rhizoferrin/100 μM FeCl_3_ after addition of Ca^2+^ (10 mM) and ascorbate (2.5 mM) and comparison to ascorbate only or Ca^2+^ only.

The effect of co-extracted metals was tested in aqueous solution with added Fe^3+^ and Mn^2+^. Rhizoferrin and protochelin (but not DFOB) signals were strongly reduced in the presence of an excess of Fe^3+^ or Mn^2+^ and became undetectable at a > 10–100 × excess (Figures 4A,B). A simple explanation would be the formation of rhizoferrin and protochelin metal complexes, but we did not observe any rhizoferrin or protochelin metal complex species (e.g., the ferric complexes) in LC-MS scans, UV-visible, or Charged Aerosol Detectors (CAD). However, addition of 2.5 mM ascorbate or 1 mM EDTA, a competitive metal chelator, to the same solution after 5 min, 1h, or 48 h, resulted in a complete reversal of the effect and peak areas were comparable to those without addition of Fe^3+^ (Figures 4A,C) or Mn^2+^. Thus, it was likely that reversible interactions of rhizoferrin and protochelin were responsible for the observed behavior.

Ascorbate functions as both a reductant and chelator (Conrad and Schade, 1968) and is effective in dissolving iron oxides by reductive dissolution (Afonso et al., 1990). Thus, any interaction of rhizoferrin or protochelin that lead to undetectable LC-MS signals could be fully reversed by the addition of a chelator, such as EDTA or a combined chelator/reductant like ascorbate. An excess of Fe^3+^ can lead to the formation of iron oxide colloids, even at low pH values (Grundl and Delwiche, 1993). These colloids may adsorb rhizoferrin and protochelin and, in effect, reduce the LC-MS signals of these siderophores (Upritchard et al., 2007; Duckworth et al., 2008). It is worth noting, that our observations with siderophores were consistent with previous studies on organic carbon in soil which indicated that carboxylated and aromatic moieties selectively bind to iron coprecipitates (Adhikari et al., 2017). Ascorbate or EDTA may have liberated rhizoferrin and protochelin in our study by dissolving iron oxide colloids (Afonso et al., 1990). Since Ca^2+^ alone had no effect (Figure 4C), this also suggested that the function of ascorbate to bind, reduce, and dissolve metal oxides may play a significant role in the synergistic activity of Ca^2+^ and ascorbate for the quantitative recovery of rhizoferrin observed in this study. As stated above, DFOB detection was not affected by an excess of metal or by the addition of Ca^2+^ and ascorbate in this context. The robust detection and recovery of the hydroxamate representative DFOB in the presence of metals, colloids, and other matrix components may be one explanation why soil siderophore analyses have frequently reported hydroxamates while α-hydroxycarboxylates or catecholates have not been detected previously.

#### Oxidation Reactions

**FIGURE 5:**
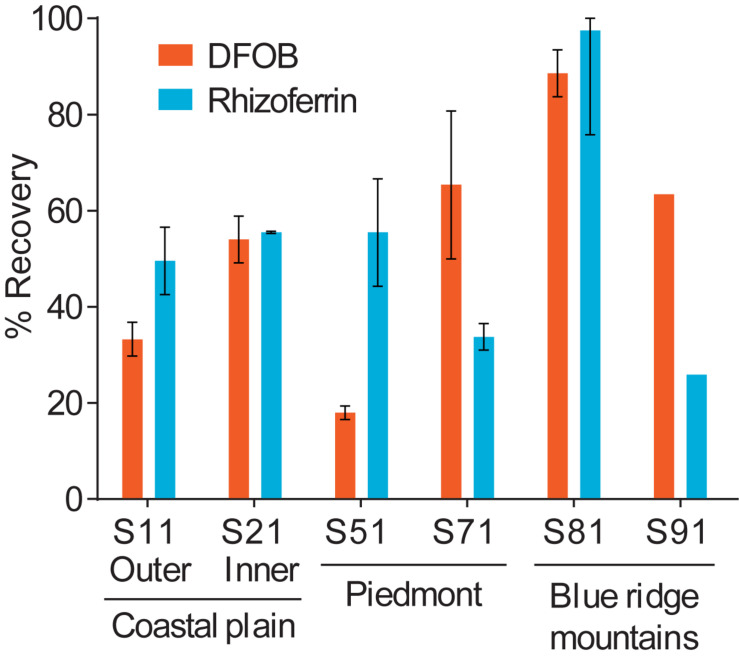
Relative LC-MS peak areas for protochelin in two experiments: (i) protochelin spiked soil was extracted under regular atmosphere (control) and low oxygen conditions (N_2_-purged solutions and headspace); (ii) protochelin standards were added to unspiked soil extracts under regular atmosphere or N_2_-purged conditions.

Catechol siderophores, including protochelin, are known to readily undergo oxidation via a reaction of the vicinal hydroxyl groups with dioxygen (Devlin and Harris, 1984; Cornish and Page, 1998; Harrington et al., 2012a). Oxidation reactions can involve initial formation of the o-quinone, followed by a subsequent reaction to cross-linked and polymeric products (Baars et al., 2015; Maier et al., 2018; Saiz-Poseu et al., 2019). To evaluate the impact of oxygen on the recoveries of protochelin, the following two experiments were set up: (i) extraction and analysis of protochelin spiked soil with the optimized method under low oxygen condition, i.e., using nitrogen-purged solutions and headspace; and (ii) analysis of protochelin standards added to unspiked soil extracts under low oxygen conditions. The results showed that protochelin signals increased in both experiments from < 2% in the presence of oxygen to ∼40% with low oxygen conditions (Figure 5). This observation suggested that protochelin recoveries and LC-MS peak areas may be low because of fast oxidation reactions. However, we found no potential protochelin oxidation products in LC-MS scans or LC-UV/vis profiles (e.g., absorption spectra characteristic of catechols or quinones) in any of the experiments in this study. An alternate explanation is that other factors, such as agglomeration of colloids related to the nitrogen purging procedure (see colloidal effects above) could be responsible for the enhanced protochelin signals in N_2_-purged experiments. Purged samples contained lower colloidal concentrations based on visibly clearer (less brown coloration) solutions and lower CAD detector baseline counts.

### Effect of Soil Chemistry on Recoveries

**FIGURE 6:**
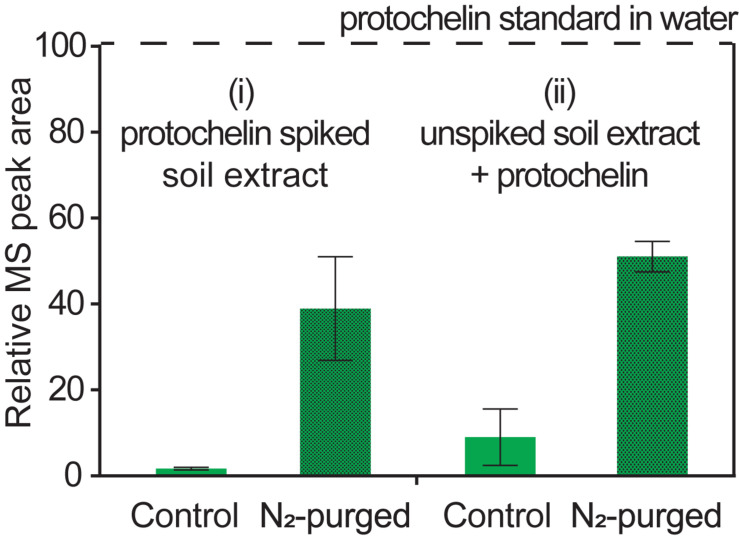
Differences in the spike-recovery using the optimized extraction method with soils collected from different physiographic regions in North Carolina. Error bars represent results from duplicate extractions.

Using the optimized extraction protocol, recoveries were evaluated with an extended range of soils with contrasting edaphic properties, including CEC, organic matter content, Fe content, and particle size distributions (Supplementary Table S1). Soils were collected across distinct physiographic regions of North Carolina. Calibration for these samples was done by internal standard additions to account for differences in sensitivity with different soil extracts. The recoveries for DFOB and rhizoferrin (Figure 6) ranged between ∼20% and full recovery. DFOB and rhizoferrin showed no statistically significant (*p* < 0.05) correlation to CEC (*R*^2^ = 0.34; *p* = 0.22 and *R*^2^ = 0.17; *p* = 0.42), organic matter (*R*^2^ = 0.20; *p* = 0.38 and *R*^2^ = 0.12; *p* = 0.51), Fe content (R^2^ = 0.45; p = 0.14 and R^2^ = 0.05; p = 0.68), or the fraction of clay (*R*^2^ = 0.24; *p* = 0.33 and *R*^2^ = 3 × 10^–6^; *p* = 0.99) (Supplementary Figure S1). There was also no significant relationship between the recovery of DFOB and rhizoferrin (*R*^2^ = 0.11; *p* = 0.52). The absence of a clear correlation between the recoveries of DFOB and rhizoferrin and soil parameters suggested that the fraction of siderophores that was not recovered with the chemical extraction method was bound to specific soil components that were not reflected by the measured bulk parameters (CEC, Fe, organic matter, particle sizes). In addition to differences in soil chemistry, microbial degradation of siderophores could have contributed to variable recoveries. Soil samples were spiked by overnight equilibration with the siderophore standards at room temperature. A previous report has demonstrated microbial degradation of the phytosiderophore 2′-deoxymugineic acid (DMA) in soil suspensions on the timescale of 8–24 h (Schenkeveld et al., 2014b).

### Analysis of Siderophores in Soil Samples

**FIGURE 7:**
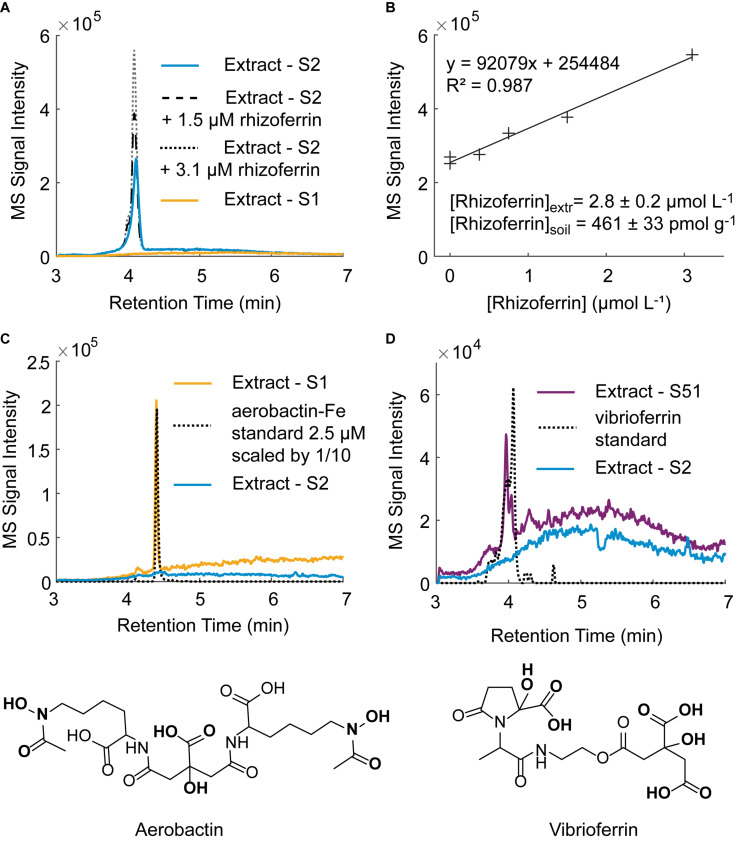
Tentative identification of siderophores in unspiked soil samples by LC-MS. **(A)** Rhizoferrin peak in soil sample S2 with two internal standard additions. A chromatogram for an extract of soil S1 in which no rhizoferrin was detected is shown for comparison. **(B)** Rhizoferrin internal standard calibration line. **(C)** Tentative identification of the aerobactin-Fe complex in soil S1 and structure of aerobactin. **(D)** Tentative identification of vibrioferrin in soil S51 and structure of vibrioferrin.

Using the optimized extraction method, we surveyed for the occurrence and concentration of siderophores in the nine soil samples used in this study (Supplementary Table S1). For the purposes of this study, a targeted detection method was set up using nine siderophore standards (DFOB, rhizoferrin, protochelin, aerobactin, vibrioferrin, amphibactin ACA, azotochelin, desferrioxamine E, pyoverdine 7.1). Each of these siderophores was previously reported to be produced by soil-dwelling microorganisms, including *Azotobacter* spp. (Baars et al., 2015; Zhang et al., 2019), *Pseudomonas* spp. (Lewis et al., 2019), and *Streptomyces* spp. (Imbert et al., 1995) but only hydroxamate siderophores, including DFOB, have been detected in soil samples (Essen et al., 2006; Ahmed and Holmstrom, 2014; Boiteau et al., 2018). Based on the nine siderophores standards, we tentatively identified three siderophores in unspiked soil extracts: rhizoferrin, vibrioferrin, and aerobactin (Figure 7). The structures of the three siderophores have an α-hydroxycarboxylate iron chelating moiety in common and represent the first detection of α-hydroxycarboxylate siderophores in field soil samples. Rhizoferrin, is characterized by three α-hydroxycarboxylate chelating groups (Figure 1). Vibrioferrin includes α-hydroxycarboxylate and carboxylate binding groups (Figure 7D), and aerobactin is a mixed α-hydroxycarboxylate/hydroxamate siderophore (Figure 7C). Rhizoferrin was detected in two top-soil samples collected from urban areas (S2, pH = 6 and S3, pH = 6.8) and near an agricultural research station (S71, pH = 6.7). Quantification of rhizoferrin by internal standard additions showed high concentrations, reaching 461 ± 33 pmol g^–1^ in S2 (Figures 7A,B). Rhizoferrin can be produced by various fungi and bacteria in soil (Crowley, 2006;Hider and Kong, 2010). Vibrioferrin (Figure 7C) was detected close to the detection limit (∼20 pmol g^–1^) in the A horizon of an acidic soil in the Piedmont region of North Carolina (S51, pH = 5). Vibrioferrin has previously been identified in cultures of the soil-dwelling N_2_-fixing bacteria *Azotobacter vinelandii* (Baars et al., 2015) and *Azotobacter chroococcum* (Zhang et al., 2019). The aerobactin-Fe complex (Figure 7D), was detected in an acidic urban soil sample (∼40 pmol g^–1^ at S1, pH = 4.8). In comparison to rhizoferrin and vibrioferrin, the aerobactin-Fe complex is stable at low pH values because of two chelating hydroxamate groups in the structure. Aerobactin was previously observed to be preferentially produced in laboratory cultures at acidic pH (pH = 5.6) by probiotic *E. coli* (Valdebenito et al., 2006). A recent study of microbial genomes in the root environment of grapevines suggests that some soil dwelling *Pseudomonas* spp. produce this siderophore (Lewis et al., 2019). While α-hydroxycarboxylates appeared to be common in soil samples, no catecholate siderophores were detected. For one replicate experiment, extractions were conducted under low oxygen conditions by purging solutions and headspace with nitrogen (see section “Oxidation Reactions”), but no signal was found for protochelin or other potential catechol siderophores.

Iron limitation is a well-recognized problem in high pH soils because of low solubility and strong interaction with particulates. Iron limitation and the role of siderophores is less clear in soils with low pH. Our results suggest that iron limitation may be prevalent even at pH values < 7, leading to microbial siderophore production. It is worth noting that all soils in our study had high soluble (< 0.2 μm) Fe concentrations (40–244 ppm), suggesting that a large fraction of iron in these soils was bound to humic matter or colloids. The observation of siderophores in these soils suggested that this soluble fraction of iron had low bio-availability. Further studies in soil are needed to test these hypotheses.

## Summary and Conclusion

Previous analyses of siderophores in soil showed the widespread presence of hydroxamate siderophores. Based on the extraction recovery experiments in this study, a bias for the recovery of hydroxamates (represented by DFOB) may explain why α-hydroxycarboxylates and catecholate siderophore were not previously detected in soil. Specifically, earlier studies targeted fungal and bacterial tris-hydroxamates by LC-MS (ferricrocin, ferrichrysin, ferrichrome) using drainage centrifugation from ∼250 g of soil (∼4–50 pmol g^–1^ of soil) (Essen et al., 2006). Ali et al. (2011) evaluated extractions with water, methanol, and K_2_HPO_4_ (1 mM, pH = 7.5) as a buffer and detected the tris-hydroxamate ferricrocin with concentrations up to 389 pmol g^–1^. Ahmed & Holmstrom extended the set to include additional fungal and bacterial hydroxamates and found up to ∼50 pmol g^–1^ in soil water and methanolic extracts (Ahmed and Holmstrom, 2014). Recently, water extracts from grassland soils revealed carboxylate phytosiderophores produced by graminaceous plants (nicotianamine, deoxymugineic acid, mugineic acid) and the fungal tris-hydroxamate ferricrocin with concentrations between 2–90 pmol g^–1^ of soil (Boiteau et al., 2018).

The extraction method developed in this study enables near quantitative recovery of not only hydroxamates (represented by DFOB) but also α-hydroxycarboxylates (represented by rhizoferrin). Application of the method revealed the first detection of α-hydroxycarboxylate siderophores in soil samples with concentrations reaching 461 pmol g^–1^, exceeding previously reported siderophore concentrations in soil. The method uses a combination of chemical mechanisms to liberate siderophores adsorbed to soil particles: an aqueous extraction with Ca^2+^ (E mechanism) and ascorbate (SA mechanism) followed by sequential re-extraction with 5%, 25%, and 80% methanol (H mechanism). The combination of Ca^2+^ and ascorbate showed a synergistic effect for the recovery of rhizoferrin. Experiments with colloids and iron additions (see sections “Protochelin Interactions With Colloidal Soil Components” and “Interaction With Dissolved Metals”) supported a mechanism in which ascorbate liberates siderophores bound to colloidal and particulate metal centers by reductive dissolution and chelation of metals, while Ca^2+^ can substitute into cation exchange sites and saturate free cation exchange sites.

In contrast to the hydroxamate and α-hydroxycarboxylate siderophores, the catecholate protochelin showed low recoveries in all conditions. The analysis was complicated by an apparent strong interaction of protochelin with colloidal soil components, such as humic matter, clay, and iron oxides (see section “Causes of Low Recoveries of the Catechol Siderophore Protochelin”). Extractions under low oxygen (N_2_-purged) conditions significantly improved recoveries, but no catechol siderophores were detected in unspiked soil samples. Based on these results, it was possible that catechol siderophores have a comparatively short lifetime in soil because of oxidation reactions (Devlin and Harris, 1984; Cornish and Page, 1998; Harrington et al., 2012a; Maier et al., 2018; Saiz-Poseu et al., 2019). In addition, catechol siderophores can be expected to strongly bind to soil particulates and may be particularly challenging to extract. The specific strong adsorption of catechol moieties to mineral and soil particles has been well established (Furubayashi et al., 2007; Maier et al., 2015; Saiz-Poseu et al., 2019). The interactions between catechols and particulate surfaces can include a combination of hydrogen bonding, coordination, π- π stacking, and covalent binding via Michael-addition following initial oxidation (Saiz-Poseu et al., 2019). Further studies focusing on the interactions of catechols with soil particle surfaces at different pH values are needed to provide insight into the lifetime and mobility of catechol siderophores in soil, and to devise specific extraction strategies.

The application of the new extraction method suggested a widespread presence of siderophores with α-hydroxycarboxylate moieties in environmental soil samples (rhizoferrin, vibrioferrin, and the mixed hydroxamate/α-hydroxycarboxylate aerobactin). Interestingly, rhizoferrin and vibrioferrin are relatively weak siderophores. Rhizoferrin has previously been shown to provide available iron to plants with strategy I (reductive iron uptake) and strategy II (exchange of iron with phytosiderophores) (Yehuda et al., 1996, 2000; Crowley, 2006). The soils in which siderophores were detected were acidic with pH values ranging between pH = 4.8–6.8. Our results suggest that even at lower pH, iron availability and siderophore production are essential for bacterial and fungal activity and become a significant control in areas of high productivity, such as plant rhizosphere environments.

The new extraction method unlocks capabilities to detect structurally diverse siderophores directly in soil samples and helps to elucidate the role of iron as a bottom-up control in soil environments. A high recovery reduces required soil amounts (0.5 – 3 g) and can enable further investigations into siderophore ‘hotspots’ and ‘hot moments’ in the heterogeneous soil environment as well as the fate of siderophores.

## Data Availability Statement

The raw data supporting the conclusions of this article will be made available by the authors, without undue reservation.

## Author Contributions

VR and OB conceived and designed experiments. VR and NF performed the experiments. VR, OB, and NF analyzed the data. OB, OD, VR, and NF wrote the manuscript. All authors read and approved the final manuscript.

## Conflict of Interest

The authors declare that the research was conducted in the absence of any commercial or financial relationships that could be construed as a potential conflict of interest.
