# Supplementary material for: Extraction and Detection of Structurally Diverse Siderophores in Soil
Source: Front Microbiol. 2020 Sep 17;11:581508. doi: 10.3389/fmicb.2020.581508 (PMC7527475; doi:10.3389/fmicb.2020.581508)
Supplement: Supplementary file 1 [file Data_Sheet_1.PDF]

Supplementary Material

1 Supplementary Figure

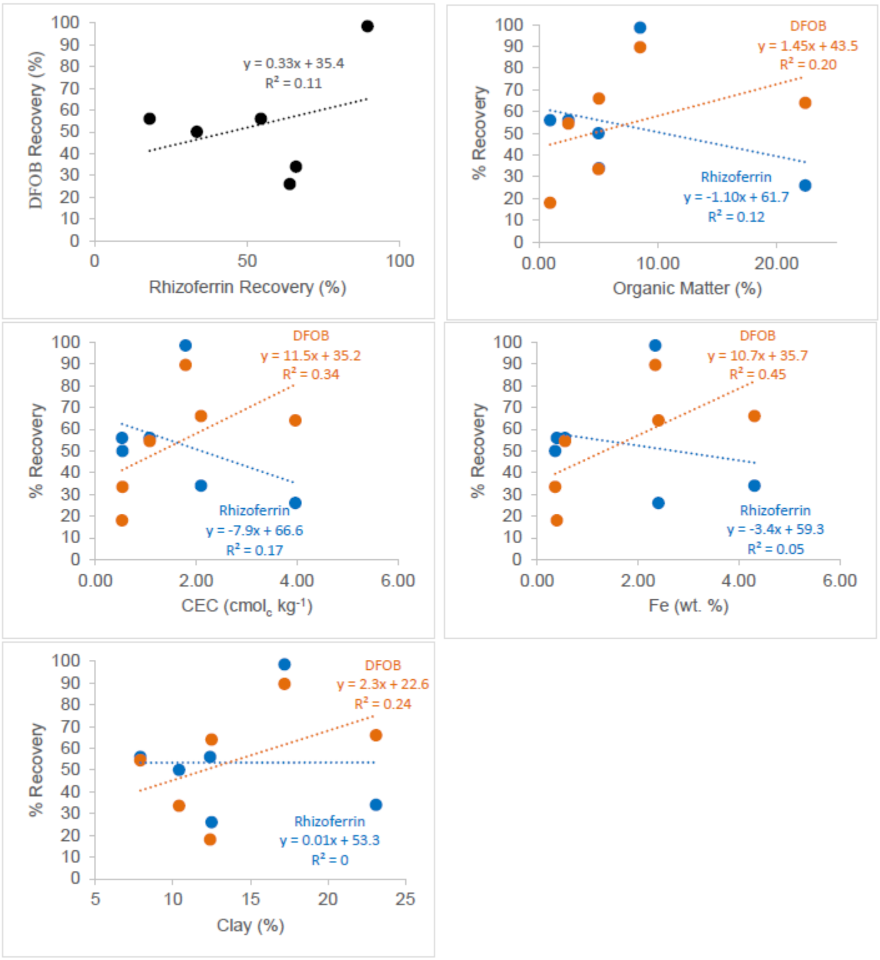

Figure S1: Correlation between DFOB and rhizoferrin recoveries with soil chemical and particle size properties

2 Supplementary Tables

Table S1: Soil edaphic properties

Sample Site	ID	Latitude, Longitude	Horizon	Texture	% sand/silt/clay	pH	% Water	% OM	% C	% N	% Fe	CEC ($cmol_c$/kg)	Water extractable metals (ppm)								% Spike recovery (duplicate analyses)	
													Fe	Al	Mn	Pb	Na	Mg	Ca	K	DFOB	Rhizo-ferrin
Outer Coastal Plain	11	35.871630, -76.657022	A	sandy loam	62/27.6/10.4	5.3	11.6	5.0			0.4	0.5	11.7	34.2	0.7	0.05	156	115	30	169	36; 31	45; 55
Inner Costal Plain - Floodplain	21	35.616502, -77.363711	A	loamy sand	84.4/7.6/7.9	7.6	8.7	2.5			0.6	1.1	32.5	45.6	0.8	0.06	381	283	726	87	51; 58	56; 56
Piedmont Triassic Basin	51	35.786454, -78.922136	A	sandy loam	60.8/26.8/12.4	5.0	9.5	1.0			0.4	0.5	40.7	36.2	0.1	0.07	42	26	18	73	17; 19	64; 48
Piedmont Research Station	71	35.688112, -80.608110	A	loam	37.8/39.2/23.1	6.7	18.6	5.1	1.3	0.1	4.3	2.1	40.3	23.7	0.5	0.02	60	1302	309	509	77; 55	32; 36
Blue Ridge Mountains	81	36.401414, -81.301119	A	loam	44.2/38.5/17.2	6.7	24.5	8.5	2.9	0.3	2.3	1.8	2.5	4.6	0.0	0.02	60	890	177	125	86; 93	83; 114
Blue Ridge Mountains	91	36.319260, -81.711270	A	sandy loam	55.2/32.2/12.5	4.3	45.8	22.4	9.6	0.9	2.4	4.0	-0.5	26.0	24	0.02	148	887	546	409	64	26
Urban Soil 1	1	35.774779, -78.674731	O	sandy loam	72.7/17.8/9.5	4.8		11.3			1.6	1.6	154	220.4	2.5	0.60	106	111	43	487		
Urban Soil 2	2	36.212040, -80.283390	O			6.0							244	128.5	1.7	0.14	731	1805	481	625		
Urban Soil 3	3	36.212040, -80.283390	O	sandy loam	59.8/23.6/16.6	6.8		9.9			1.0	1.8	121	121.1	3.1	0.09	160	1031	394	1163		

Table S2: Siderophore standards, ionization mode, and *m/z* values used for tentative identification of siderophores in unspiked soil samples by LC-MS.

**Siderophore**	**Ionization**	**Exact mass (M)**	***m/z* (MH^+)**	***m/z* $(M\text{-}H)^-$**	***z***
Aerobactin - Fe complex	Positive	617.1394	618.1		1
Amphibactin ACA	Positive	791.4271	792.4		1
Desferrioxamine B	Positive	560.3534	561.4		1
Desferrioxamine B - Fe complex	Positive	613.2648	614.3		1
Desferrioxamine E - Fe complex	Positive	653.2597	654.3		1
Pyoverdine 7.1	Positive	1046.4901	523.7		2
Pyoverdine 7.1 - Fe complex	Positive	1099.4016	550.2		2
Azotochelin	Negative	418.1376		417.1	1
Protochelin	Negative	624.2431		623.2	1
Vibrioferrin	Negative	434.1173		433.1	1
Rhizoferrin	Negative	436.1329		435.1	1
